# The Language We Use to Teach Surgery: Language Inaccessibility of Minimally Invasive Surgical Training in Francophone Countries

**DOI:** 10.1002/wjs.12656

**Published:** 2025-06-13

**Authors:** Callie K. VanWinkle, Tchinde Ngueping Marius Jordan, Blessing Ngam, Keir Thelander, Grace J. Kim

**Affiliations:** ^1^ University of Michigan Medical School University of Michigan Ann Arbor Michigan USA; ^2^ Mbingo Baptist Hospital Bamenda Cameroon; ^3^ Pan‐African Academy of Christian Surgeons Palatine Illinois USA; ^4^ Loma Linda University Loma Linda California USA; ^5^ Department of Surgery University of Michigan Ann Arbor Michigan USA

**Keywords:** francophone, laparoscopic surgery, minimally invasive surgery, surgical education

Minimally invasive surgery was pioneered in Europe in the early 20th century, with the first recorded laparoscopic cholecystectomy documented in 1987 by a French surgeon Phillip Mouret [1]. Although laparoscopy has now been widely adopted globally, its uptake in low‐ and middle‐income countries has been variable despite clinical advantages and interest from surgeons [2]. Commonly cited barriers include financial constraints, access to required equipment, and availability of training opportunities [2, 3]. However, literature assessing the role of language heterogeneity as a barrier to adopting laparoscopy has been limited.

A 2023 paper found 39.1% of countries rely on materials in foreign languages in medical education. Among continents, Africa showed the highest dependence on foreign languages in medical education [4]. We specifically investigated the language accessibility of laparoscopic training materials on the African continent, where colonialism has profoundly influenced the linguistic landscape. Of the estimated 300 million French language speakers around the world, 44% live in Africa and 85% are projected to be living in Africa by 2050 [5]. Acknowledgment of this foothold of the French language on education in Francophone Africa is increasingly important.

Laparoscopic training in Francophone Africa is currently limited [2]. Trainees often rely on a combination of in‐person training through international programs and supplemental online training resources [2, 6]. International training programs often necessitate that trainees travel from low‐income countries (LICs) to high‐income countries (HICs) [6]. Although this approach may offer many benefits, its deficits have also been widely discussed in literature [6, 7]. “Brain drain,” the phenomenon of skilled workers emigrating from LICs to HICs, has been cited as one such possible challenge [6]. Although recent literature suggests the effects of “brain drain” may not be as substantial as previously thought in the evolving context of the globalization of surgery, other pitfalls persist [6, 7]. Travel costs and program enrollment fees can be prohibitive and place undue burden on trainees to leave their home and learn in settings vastly different from their intended future practice environment [6]. Another concern is the tendency of these programs to prioritize HIC trainees over LIC trainees, termed “surgical colonialism” [6]. Finally, surgical training is an iterative learning process that extends well beyond a surgical apprenticeship completed abroad. Sustained access to resources, including texts, videos, simulations, and mentorship, is critical for the continued refinement of a surgeon's knowledge and skills and even more so in minimally invasive surgical techniques [3]. In more recent years, to address the limitations of the international in‐person training schema, several working groups have sought to address this gap in access to laparoscopy training through the development of online training materials, ranging from reference textbooks to video‐based learning and longitudinal technical skills development and mentorship [8, 9].

Despite the French origins of laparoscopic abdominal surgery, few online open‐source laparoscopy training materials are available in the French language. When gauging the utility of educational resources for surgical trainees with otherwise limited access to laparoscopic training, the accessibility of the resource is impacted significantly by the user's fluency in the language of the training material. Yet, commonly used resources have produced few, if any, materials in French [8, 10]. Even those resources with French‐translated user interfaces still feature videos narrated in English [8]. Scarcity of language‐accessible versions of these training resources hinders Francophone surgical trainees in LICs from establishing safe and efficacious practices in minimally invasive surgery.

One initiative addressing this gap is ALL‐SAFE, a program developed to support surgical training courses teaching the cognitive and psychomotor skills needed to perform laparoscopic surgery in a low‐resource setting. In 2024, ALL‐SAFE began to offer new modules in the French language in addition to English language [9]. This initiative was prompted by multiple requests from surgeons in Francophone Africa for materials in French to support surgical learning. This effort was also supported by a teaching trip to Conakry, Guinea, where a laparoscopic course was conducted entirely in the French language. Thus, the new French‐translated ALL‐SAFE modules fill a notable gap in existing resources, particularly on the African continent where a high degree of language variance exists in medical education. As more material becomes readily available in the French language, the utility and efficacy of laparoscopic training materials for Francophone Africa needs to be studied.

We urge our colleagues in surgical education to address this critical gap in minimally invasive surgery training created by dominance of the English language in current training materials. Minimally invasive surgery is here to stay, and many of its aspiring users primarily speak French.

## Author Contributions

**Callie K. VanWinkle:** writing – original draft, conceptualization, writing – review and editing. **Tchinde Ngueping Marius Jordan:** writing – review and editing. **Blessing Ngam:** writing – review and editing. **Keir Thelander:** writing – review and editing, conceptualization. **Grace J. Kim:** conceptualization, writing – review and editing, supervision.

## Conflicts of Interest

The authors declare no conflicts of interest.

## Data Availability

Data sharing is not applicable to this article as no new data were created or analyzed in this study.
